# Enteral nutrition with eicosapentaenoic acid, γ-linolenic acid and antioxidants in the early treatment of sepsis: results from a multicenter, prospective, randomized, double-blinded, controlled study: the INTERSEPT Study

**DOI:** 10.1186/cc10267

**Published:** 2011-06-09

**Authors:** Alessandro Pontes-Arruda, Laércia Ferreira Martins, Samya Maria de Lima, Alexandre Marini Isola, Diogo Toledo, Ederlon Rezende, Marcelo Maia, Gisele Brocco Magnan

**Affiliations:** 1 Department of Nutrition and Intensive Care, Fernandes Távora Hospital, Avenida Francisco Sá, 5445, Fortaleza, Ceará, 60.30-002, Brazil; 2 Department of Intensive Care, Hospital do Servidor Público Estadual de São Paulo, Rua Pedro de Toledo, 1800, São Paulo, 04.039-901, Brazil; 3 Department of Intensive Care, Hospital Santa Luzia, SHLS 716, Conjunto E, Brasília, Distrito Federal, 70.390-903, Brazil

## Abstract

**Introduction:**

Enteral nutrition (EN) with eicosapentaenoic acid (EPA)/γ-linolenic acid (GLA) is recommended for mechanically ventilated patients with severe lung injury. EPA/GLA has anti-inflammatory benefits, as evidenced by its association with reduction in pulmonary inflammation, improvement in oxygenation and improved clinical outcomes in patients with severe forms of acute lung injury. This study was a prospective, multicenter, randomized, double-blinded, controlled trial designed to investigate whether EPA/GLA could have an effective role in the treatment of patients with early sepsis (systemic inflammatory response syndrome with confirmed or presumed infection and without any organ dysfunction) by reducing the progression of the disease to severe sepsis (sepsis associated with at least one organ failure) or septic shock (sepsis associated with hypotension despite adequate fluid resuscitation). Secondary outcomes included the development of individual organ failure, increased ICU and hospital length of stay, need for mechanical ventilation and 28-day all-cause mortality.

**Methods:**

Randomization was concealed, and patients were allocated to receive, for seven days, either an EPA/GLA diet or an isocaloric, isonitrogenous control diet not enhanced with lipids. Patients were continuously tube-fed at a minimum of 75% of basal energy expenditure × 1.3. To evaluate the progression to severe sepsis and/or septic shock, daily screening for individual organ failure was performed. All clinical outcomes were recorded during a 28-day follow-up period.

**Results:**

A total of 115 patients in the early stages of sepsis requiring EN were included, among whom 106 were considered evaluable. Intention-to-treat (ITT) analysis demonstrated that patients fed the EPA/GLA diet developed less severe sepsis and/or septic shock than patients fed the control diet (26.3% versus 50%, respectively; *P *= 0.0259), with similar results observed for the evaluable patients (26.4% versus 50.9% respectively; *P *= 0.0217). The ITT analysis demonstrated that patients in the study group developed cardiovascular failure (36.2% versus 21%, respectively; *P *= 0.0381) and respiratory failure (39.6% versus 24.6%, respectively; *P *= 0.0362) less often than the control group. Similarly, when considering only the evaluable patients, fewer patientsdeveloped cardiovascular failure (20.7% versus 37.7%, respectively; *P *= 0.03) and respiratory failure (26.4% versus 39.6%, respectively; *P *= 0.04). The percentage of patients fed the EPA/GLA diet requiring invasive mechanical ventilation was reduced compared with controls (ITT patients: 18.9% versus 33.9%, respectively; *P *= 0.394; evaluable patients: 17.5% versus 34.5%, respectively; *P *= 0.295). Patients nourished with the EPA/GLA diet remained in the ICU fewer days than the control population (ITT patients: 21.1 ICU-free days versus 14.7 ICU-free days, respectively; *P *< 0.0001; evaluable patients: 20.8 ICU-free days versus 14.3 ICU-free days, respectively; *P *< 0.0001) and fewer days at the hospital (ITT patients: 19.5 hospital-free days versus 10.3 hospital-free days, respectively; *P *< 0.0001; evaluable patients: 19.1 hospital-free days versus 10.2 hospital-free days, respectively; *P *< 0.001) (all numbers expressed as means). No significant differences in 28-day all-cause mortality were observed (ITT patients: 26.2% EPA/GLA diet versus 27.6% control diet, respectively; *P *= 0.72; evaluable: 26.4 EPA/GLA diet versus 30.18 control diet, respectively; *P *= 0.79).

**Conclusions:**

These data suggest that EPA/GLA may play a beneficial role in the treatment of enterally fed patients in the early stages of sepsis without associated organ dysfunction by contributing to slowing the progression of sepsis-related organ dysfunction, especially with regard to cardiovascular and respiratory dysfunction.

**Trial Registration:**

ClinicalTrials.gov: NCT00981877

## Introduction

It has been demonstrated that nutritional support by feeding patients specialized diets, as a result of its capacity to interfere with a variety of biological processes, can modulate the chain of inflammatory responses [1-3]. Recent pharmaceutical interventions proposed for sepsis have sought to focus on regulating the chain of pro- and anti-inflammatory mediators [4,5], which are responsible for causing the systemic characteristics of the disease and consequently leading to multiple organ failure. The inflammatory reaction is capable of activating the synthesis of several lipid mediators which are involved in the complex regulation of the inflammatory process [6].

Lipid mediators are synthesized by three main pathways, cyclooxygenase, 5-lipoxygenase and cytochrome P450, by using fatty acids such as arachidonic acid (AA), eicosapentaenoic acid (EPA) and γ-linolenic acid (GLA) as substrates [7], but the biological anti-inflammatory activities of EPA and GLA are far beyond the simple regulation of eicosanoid production. For instance, EPAs can affect immune cell responses through the regulation of gene expression and subsequent downstream events by acting as ligands for nuclear receptors [8] and as control transcription factors [9]. EPA can also affect the activity of the proinflammatory transcription nuclear factor κB (NF-κB), which regulates the expression of many proinflammatory gene-encoding adhesion molecules, cytokines, chemokines and other effectors of the innate immune response system [10]. Researchers have recently described that the two main active fish oil pharmaconutrients, EPA and docosahexaenoic acid, are substrates of two novel classes of mediators called resolvins and protectins [11,12], which are involved in the resolution of the inflammatory process [13-15].

In 1999, Gadek and co-workers [16] demonstrated that the use of a diet enriched with EPA, GLA and antioxidants can improve oxygenation status in patients with acute respiratory distress syndrome (ARDS). The same study demonstrated that patients nourished with this diet spend fewer days in the ICU as well as in the hospital. This diet was further evaluated in two additional studies published in 2006 [17,18]. Singer *et al. *[17] demonstrated the effectiveness of an EPA/GLA diet in improving oxygenation status and decreasing the ICU and hospital length of stay (LOS) of patients with acute lung injury (ALI). This diet was also associated with lower mortality rates on the basis of 28-day all-cause mortality. Similar results were observed by Pontes-Arruda *et al. *[18] in which such a diet was fed to patients with ARDS secondary to severe sepsis and/or septic shock.

In a recently published meta-analysis of outcomes, the three above-mentioned studies were combined [19]. For the patients considered evaluable (*n *= 296), the use of an EPA/GLA diet was associated with a 60% reduction in the risk of 28-day in-hospital all-cause mortality (odds ratio (OR) = 0.40, 95% confidence interval (95% CI) = 0.24 to 0.68; *P *= 0.001). With regard to the effects of the use of an EPA/GLA diet upon mortality on the basis of intent-to-treat (ITT) analysis (*n *= 411 patients), a 49% reduction in the risk of 28-day in-hospital all-cause mortality was evident (OR = 0.51, 95% CI 0.33 to 0.79; *P *= 0.002). Available clinical evidence is also consistent in showing a reduction in time on mechanical ventilation as well as ICU and hospital LOS. In fact, a second meta-analysis including data from unpublished studies [20] demonstrated similar results.

In analyzing the data together, it was clear that feeding patients an EPA/GLA diet was associated with a reduction in the development of new organ dysfunction. Although the Singer *et al. *study [17] did not assess this variable, both Gadek *et al. *[16] and Pontes-Arruda *et al. *[19] demonstrated reductions in this particular outcome. Combined, the feeding patients an EPA/GLA diet was associated with an 83% reduction in the development of new organ failure. If we accept that the development of new organ failure is the pathway that leads patients with sepsis to severe sepsis and septic shock, it appears logical to evaluate the possible benefits of treating patients with this nutritional intervention in the early stages of sepsis (defined as systemic inflammatory response syndrome associated with confirmed or presumed infection and without any organ failure) as a way to prevent the evolution or to slow progression of the disease. Moreover, if we consider that the development of multiple organ failure is associated with increased mortality rates, we can hypothesize that feeding patients a diet including an enteral formulation enriched with EPA/GLA might be a determining factor in reducing the mortality rate.

All previously published trials were performed in critically ill, mechanically ventilated patients with at least one organ failure. Since the effects of the EPA/GLA diet in patients without any organ failure remain uncertain, the aims of this clinical study were to evaluate the role of an enteral formulation enriched with EPA/GLA in patients diagnosed in the very early stages of sepsis, despite respiratory failure, and to compare the results with those obtained with the use of a standard ICU formulation, isocaloric and isonitrogenous to the study diet, and not enhanced with lipids but higher in carbohydrates.

## Materials and methods

### Study design and ethical aspects

In this prospective, multicenter, randomized, double-blinded, controlled trial, we compared the effects of feeding patients with early sepsis a diet including enteral nutrition enriched with EPA, GLA and elevated levels of antioxidant vitamins (Oxepa™; Abbott Nutrition, Chicago, IL, USA) those of an isonitrogenous and isocaloric control diet (Ensure Plus HN™; Abbott Nutrition, Chicago, IL, USA). The control diet used did not contain EPA or GLA, but was higher in carbohydrates than the study diet and therefore was considered a standard ICU diet. The complete composition of the diets fed to the patients is given in Table 1. The study was approved by 12 centers in Brazil, and the patients were recruited from five of these centers during a 21-month period from 10 July 2007 to 27 April 2009.

**Table 1:** Composition of the study and control diets used

Diet components	Study diet (Oxepa™)^a^	Control diet (Ensure Plus HN™)^a^
Protein		
Percentage of total calories	16.7	16.7
Concentration, g/L	62.5	62.7
Sources	87% sodium caseinate 13% calcium caseinate	73% sodium caseinate 11% calcium caseinate 16% soy protein isolate
Carbohydrates		
Percentage of total calories	28.1	54.3
Concentration, g/L	105.5	204
Sources	45% maltodextrin 55% sucrose	100% maltodextrin
Lipids		
Percentage of total calories	55.2	29.0
Concentration, g/L	93.7	49.1
Sources	31.8% canola oil 25% medium chain triglycerides 20% borage oil 20% fish oil 3.2% soy lecithin	50% high oleic safflower oil 30% canola oil 20% medium chain triglycerides
Ω-6:Ω-3 ratio	1.85:1	3.8:1
Ω-3, g/L	10	1.5
Eicosapentaenoic acid (EPA), g/L	4.5	0
γ-Linolenic acid (GLA), g/L	4.3	0
Docosahexaenoic acid (DHA), g/L	2.0	0
Vitamins		
E, IU/L	320	39
C, mg/L	840	130
β-carotene, mg/L	670	345
Taurine, mg/L	320	150
L-carnitine, mg/L	120	120
A, IU/L	12,000	4,167
D, IU/L	430	400
K_1_, μg/L	100	80
Folic acid, μg/L	420	400
Thiamine, mg/L	3.2	2.8
Riboflavin, mg/L	3.6	3.4
B_6_, mg/L	4.3	3.9
B_12_, mg/L	6.0	4.0
Niacin, mg/L	29	29
Choline, mg/L	640	800
Biotin, μg/L	1	72
Pantothenic acid, mg/L	13	13
Trace minerals		
Na, mg/L	1,310	1,400
K, mg/L	1,960	1,650
Cl, mg/L	1,690	1,450
Ca, mg/L	1,060	1,000
P, mg/L	1,000	1,000
Mg, mg/L	320	310
I, μg/L	160	150
Mn, mg/L	5.4	5.0
Cu, mg/L	2.2	2.5
Zn, mg/L	18	19
Fe, mg/L	20	22
Se, μg/L	130	70
Cr, μg/L	160	100
Mo, μg/L	130	150
Caloric density, kcal/mL	1.5	1.5
Osmolarity, mOsmol/L	385	392

^a^Oxepa™ and Ensure Plus HN™ are trademarked products of Abbott Laboratories (Chicago, IL, USA).

This study was approved by the institutional review boards of each participating institution, by the Brazilian Federal Commission of Ethics and Research (CONEP) and by the Brazilian National Agency of Sanitary Vigilance (ANVISA), as required by law, before any patient enrollment. Informed written consent was obtained from all patients or from their legal representatives. This study was performed in accordance with all ethics statements in the Declaration of Helsinki (52nd General Assembly of the World Medical Association, Edinburgh, UK, October 2000) and the Nuremberg Code [21]. All included patients were treated in accordance with the Surviving Sepsis Campaign guidelines for the management of severe sepsis and septic shock [22], which means that adequate initial resuscitation was administered to all patients with sepsis-induced tissue hypoperfusion, in accordance with the protocol described by Rivers *et al. *[23], and broad spectrum antibiotics were administered within the first hour of ICU admission.

### Study patients

The study inclusion criteria were that patients had to be older than 18 years of age, at the ICU with a clinical diagnosis of early sepsis and required enteral nutrition. A 36-hour window was allowed to establish a clinical diagnosis of sepsis, obtain the necessary written informed consent from the patients and randomize the patients. The diagnosis of sepsis was made on the basis of the criteria previously defined by Bone *et al. *[24] and as modified by Bernard *et al. *[25]. The following were considered exclusion criteria: severe sepsis (sepsis associated with at least one organ failure) and septic shock (sepsis associated with hypotension despite adequate fluid resuscitation) at baseline, a clinical diagnosis of sepsis for more than 36 hours, pregnancy or breastfeeding, patients younger than 18 years of age, significant limitation of survival prognosis (patients expecting with a life survival expectancy less than 28 days because of a chronic and/or incurable disease such as uncontrolled cancer or other terminal disease), preexisting chronic renal insufficiency and need of hemodialysis or peritoneal dialysis, body mass index (BMI) ≥ 29, acute pancreatitis without established origin, participation in other clinical trials less than 6 months before this trial, head trauma with a Glasgow Coma Scale score ≤ 5, recent stroke or subarachnoid hemorrhage (less than 3 months), severe immunologic suppression (defined as a leukocyte count below 5,000 cells/mm^3^), HIV infection, no indication for enteral feeding or imminent need for parenteral nutrition, partial parenteral nutrition to achieve caloric goals, presence of uncontrolled diarrhea, recent gastrointestinal bleeding, physician's decision to exclude patients on the basis of the study protocol, or the presence of respiratory failure (defined as evidence of acute pulmonary dysfunction with a ratio of partial pressure of oxygen to fraction of inspired oxygen (PaO_2_/FiO_2 _ratio) < 300 and, if measured, pulmonary capillary wedge pressure not suggestive of central volume overload). Mechanical ventilation at baseline *per se *was not considered a reason for exclusion as long as the patient maintained a PaO_2_/FiO_2 _ratio not indicative of respiratory failure.

### Randomization and study diet administration

**Figure 1:**
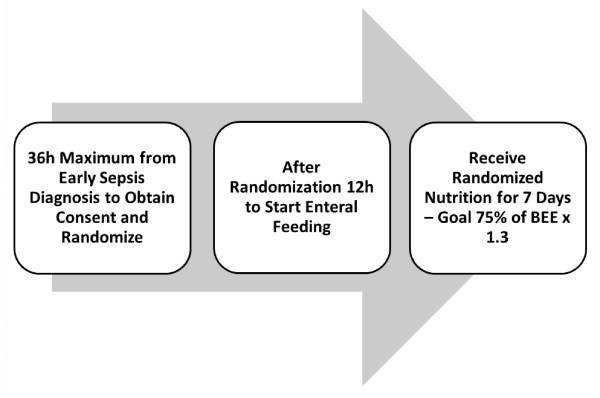
**Participant flow diagram**. BEE = basal energy expenditure.

Patients were randomized at a ratio of 1:1 using a web-based central randomization system. Patients, families and caregivers were blinded to the type of diet being delivered. All data were inserted into a web-based clinical research form located in a secure server using an encrypted and dedicated website. Randomization was performed using blocks of randomization. Each block used an algorithm with different sizes and sequences of randomization to guarantee an adequate balance between the two study groups at any time, independently of the number of patients enrolled. The caloric goal was automatically calculated by using the Harris Benedict Equation × 1.3, taking into consideration the information inserted (such as age, weight and height). After randomization, the group information (study or control) and the dietary caloric goals (measured in kilocalories per liter or in milliliters) were sent by personal email message to the nonblinded person in the center in which the patient was included. Once the patient was randomized, a 12-hour window was allowed to start the enteral diet; therefore, each institution had a 48-hour window within which to begin the study protocol including the 36 hours required to identify the patient, obtain the patient's written informed consent and randomize the patient, with an additional 12 hours to start the diet) (see Figure 1). Information was inserted daily regarding the exact date and time when the diet started as well as the patient's daily caloric intake. Patients were excluded from the trial on the basis of a protocol violation if the enteral diet was not started within 12 hours after randomization.

Study and control diets were similar in consistency and color. To maintain adequate blinding, the diets were poured into similar and sealed containers before being delivered to the ICU. The containers were connected to an infusion pump (Patrol Enteral Feeding Pump; Abbott Nutrition, Chicago, IL, USA) using a closed system to prevent any detection of the diet smell by the blinded subjects. Enteral feeding was delivered at a constant rate to achieve a minimum of 50% basal energy expenditure (BEE) × 1.3 within the first 24 hours. If well tolerated, enteral nutrition was advanced to achieve a minimum of 75% of BEE × 1.3 within 72 hours. Diets were delivered continuously without night pause and using the same protocol in all participating institutions. The enteral diets were delivered for a total of seven days or until they were interrupted at the physician's discretion or because of the development of any adverse event that could be related to the enteral feeding. The diet could also be interrupted if there was an indication to start oral feeding or parenteral nutrition. After the diet was interrupted, and if the patient was still in need of enteral nutrition, each individual institution was allowed to freely select the most appropriate diet to continue the enteral nutrition therapy. The day on which patients started receiving the diet was considered study day 1.

### Laboratory data, outcome measures and statistical analysis

Blood samples were obtained for routine laboratory testing on a daily basis. Severity scores, including the Acute Physiology and Chronic Health Evaluation II (APACHE II) score and the Sequential Organ Failure Assessment score (SOFA), were automatically calculated by the web-based system, taking into consideration the individual values inserted for each patient to avoid possible human errors in the calculations of the severity scores.

The primary issue was to determine the development of severe sepsis and septic shock during a 28-day follow-up period. To achieve this goal, a daily evaluation regarding the development of individual organ failure was performed. The criteria used to define each organ failure are given in Table 2. If the patient developed at least one organ failure, the patient was considered to have developed severe sepsis. If refractory hypotension was identified, the patient was counted as having developed septic shock. Secondary issues included ICU and hospital LOS, mechanical ventilation support, development of individual organ failure and 28-day all-cause mortality.

**Table 2:** Criteria used to define individual organ dysfunction^a^

Type of dysfunction	Definition
Cardiovascular	Systolic arterial pressure ≤ 90 mmHg after adequate fluid resuscitation
Respiratory	PaO_2_/FiO_2 _ratio ≤ 300 and, if measured, pulmonary capillary wedge pressure not suggestive of central volume overload
Hematologic	Platelet count ≤ 100,000/mm^3 ^or 50% drop in the past 72 hours or relevant changes in coagulation profile (INR > 1.5 or aPTT > 60 seconds)
Renal	Urine output < 0.5 mL/kg/hour for at least 2 hours, even after fluid resuscitation or creatinine > 2.0 mg/dL
Metabolic	pH < 7.30 or base excess less than -5 mEq/L with plasma lactate > 1.5 × normal value
Hepatic	Total bilirubin > 2.0 mg/dL or 35 mmol/L

^a^PaO_2_/FiO_2 _ratio = ratio of partial pressure of oxygen to fraction of inspired oxygen; INR = International Normalized Ratio; aPTT = activated partial thromboplastin time.

At the conclusion of the study, the whole database and the source documentation were independently audited to ensure the quality of the information collected, and the database was analyzed by an independent statistical company (Bioestatística, Montreal, QC, Canada). Categorical variables were compared between the two groups using the χ^2 ^test or Fisher's exact test as appropriate [26]. Quantitative normally distributed variables between the groups were compared using an unpaired two-sample *t*-test. For quantitative non-normally distributed data, the nonparametric Wilcoxon rank-sum test was used [26]. Normality was assessed by using the Shapiro-Wilk test [26,27]. Normally distributed data are reported as means with standard deviations (SDs), and non-normally distributed data are expressed as medians and interquartile ranges (IQRs, 25th to 75th percentiles). Categorical variables are expressed as numbers and percentages.

Estimates of survival curves during a 28-day follow-up period were calculated according to the Kaplan-Meier product limit method [28] and compared by using the log-rank test [29]. All statistical analyses were performed using SAS version 9.2 software (SAS Institute, Inc., Cary, NC, USA). All *P *values are two-tailed. *P *< 0.05 was considered statistically significant.

## Results

### Patients and demographic characteristics

A total of 115 patients were included, but nine patients (four in the study group and five in the control group) were excluded on the basis of protocol violation because their diet was not started within the 12-hour window, leaving 106 patients to be evaluated: 53 in the study group and 53 in the control group. The population of patients included was considered well balanced in terms of baseline demographic characteristics, including severity scores. The patients' median ages were 70 (IQR 64 to 78) years in the study group and 72 (IQR 65 to 82) years in the control group (*P *> 0.05), with a slightly higher number of males than females in both groups. The severity scores were considered quite high for both populations on the basis of APACHE II scores (median 19.5 (IQR 17 to 25) in the study group versus 20 (IQR 16 to 23) in the control group), SOFA scores (median 5.5 (IQR 4 to 9) in the study group versus 6 (IQR 4 to 8) in the control group) and Multiple Organ Dysfunction Score (median 4 (IQR 3 to 6) for the study group and 4 (IQR 3 to 5) for the control group) (all *P *> 0.05). Included patients also presented similar numbers of baseline comorbidities. The baseline population characteristics of both groups are given in Table 3.

**Table 3:** Baseline demographic characteristics of the patients^a^

Characteristics	Study (*n *= 53)	Control (*n *= 53)	*P *value
Age, years^b^	70 (64 to 78)	72 (65 to 82)	0.3729
Sex			
Males, *n *(%)	32 (60.4)	29 (54.7)	0.6826
Females, *n *(%)	21 (39.6)	24 (45.3)	0.7726
Weight, kg	59.3 ± 12.5	61.2 ± 11.7	0.4243
Height, cm	166.2 ± 11.4	167.7 ± 10.4	0.4749
APACHE II score^b^	19.5 (17 to 25)	20 (16 to 23)	0.5291
SOFA score^b^	5.5 (4 to 9)	6 (4 to 8)	0.7991
MODS score^b^	4 (3 to 6)	4 (3 to 5)	0.6106
Lactate, mmol/L^b^	3.75 (2.9 to 4.65)	3.7 (2.8 to 4.3)	0.7181
Blood glucose, mg/dL^b^	103 (89 to 149)	102 (87 to 125)	0.6109
Temperature, °C^b^	39.4 (38.2 to 39.8)	38.9 (37.2 to 39.5)	0.0780
GCS score	10.1 ± 2.2	10.8 ± 2.1	0.1661
White blood cells, 1 × 10^9 ^cells/L	19.3 ± 0.9	20.7 ± 0.7	0.4187
Platelets, 1 × 10^9 ^cells/L^b^	198 (144 to 259)	211 (178 to 256)	0.3580
Serum creatinine, mg/dL^b^	1.15 (0.9 to 1.6)	0.9 (0.8 to 1.3)	0.9088
Total bilirubin, mg/dL^b^	0.9 (0.7 to 1.3)	0.8 (0.7 to 1.5)	0.7739
Serum Na, mEq/L^b^	139 (133 to 145)	135 (137 to 144)	0.9053
Serum K, mEq/L	3.9 ± 0.7	4.0 ± 0.8	0.6718
Patients supported with MV, *n *(%)^c^	1 (1.9)	2 (3.8)	0.5172
Patients treated with sedatives, *n *(%)	3 (5.7)	5 (9.4)	0.1280
Baseline comorbidities, n (%)			
Recent surgery (< 3 months)	6 (11.3)	9 (16.9)	0.4008
History of alcoholism	10 (18.9)	8 (15.1)	0.6027
Asthma	12 (22.6)	17 (32.1)	0.2705
Diabetes	10 (18.9)	9 (16.9)	0.7988
COPD	11 (20.75)	12 (22.6)	0.8122
Cancer	4 (7.5)	4 (7.5)	0.6425
Thromboembolism	1 (1.9)	1 (1.9)	0.7525
History of illicit drug use	4 (7.5)	2 (3.8)	0.6425
Primary source of infection, *n *(%)			
Lungs	38 (71.7)	33 (62.3)	0.2893
Abdomen	6 (11.3)	8 (15.1)	0.5311
Urinary tract	4 (7.6)	2 (3.7)	0.1980
Other	5 (9.4)	10 (18.9)	0.0815

^a^APACHE II = Acute Physiology and Chronic Health Evaluation II; SOFA = Sequential Organ Failure Assessment; MODS = Multiple Organ Dysfunction Score; GCS = Glasgow Coma Scale; MV = mechanical ventilation; COPD = chronic pulmonary obstructive disease. Data are presented as medians (interquartile range, 25th to 75th percentiles), and *P *values were calculated using the Wilcoxon rank-sum test. ^b^Distributions considered non-normal on the basis of the Shapiro-Wilk test. ^c^All MV patients were supported with noninvasive mechanical ventilation.

### Dietary intake

The nutritional status of all patients was evaluated by using the Subjective Global Assessment (SGA) at baseline. The majority of the population included was considered to have some degree of malnourishment at baseline on the basis of the SGA. Proportions of 62.3% of the patients in the study group and 52.8% of the patients in the control group were considered either moderately undernourished or severely undernourished, but the differences between the groups in terms of nutritional status were not considered to be of statistical significance. There were no significant differences in terms of calculated caloric goals, daily volume in milliliters and total caloric intake per day. The study group received more lipids than the control group (mean 96.0 ± 37.2 g/day in the study group versus 49.8 ± 19.0 g/day in the control group; *P *< 0.0001), whereas the control population received more carbohydrates than the study group (mean 205.6 ± 77.4 g/day versus 104.2 ± 38.9 g/day; *P *< 0.0001). The nutritional parameters and dietary intake data are given in Table 4.

**Table 4:** Nutritional parameters of the included patients^a^

Variable	Study (*n *= 53)	Control (*n *= 53)	*P *value
SGA nutritional status, *n *(%)			
Well-nourished	20 (37.7)	25 (47.2)	0.4672
Moderately undernourished	22 (41.5)	21 (39.6)	0.7983
Severely undernourished	11 (20.8)	7 (13.2)	0.6974
Type of enteral feeding, *n *(%)			
Gastric	25 (47.2)	23 (43.4)	0.5112
Duodenal/jejunal	19 (35.8)	21 (39.6)	
Gastrostomy	6 (11.3)	5 (9.4)	
Jejunostomy	3 (5.7)	4 (7.6)	
Daily nutritional values			
Days receiving diet, *n*	6.09 ± 0.4	6.17 ± 0.8	0.4512
Calculated caloric goal, kcal/day^b^	1,557 (1,367 to 1,812)	1,560 (1,422 to 1,820)	0.4827
Calculated caloric volume, mL/day^b^	1,038 (911 to 1,208)	1,040 (948 to 1,213)	0.9600
Calories, kcal/day^b^	1,538 (1,295 to 1,890)	1,523 (1,370 to 1,950)	0.3974
Volume, mL/day^b^	1,025 (863 to 1,260)	1,015 (913 to 1,300)	0.3533
Lipids, g/day	96.04 ± 37.2	49.8 ± 19.0	< 0.0001
Carbohydrates, g/day	104.2 ± 38.9	205.6 ± 77.4	< 0.0001
Proteins, g/day	63.4 ± 22.8	62.6 ± 24.3	0.3188
EPA, g/day	4.6 ± 1.8	0	< 0.0001
GLA, g/day	4.4 ± 1.7	0	< 0.0001
DHA, g/day	2.05 ± 0.7	0	< 0.0001
Time to start enteral feeding, hours	8.4 ± 2.6	7.6 ± 3.1	0.2833
Time to reach 75% of caloric goal, hours	35.2 ± 8.7	32.0 ± 6.4	0.0871

^a^SGA = Subjective Global Assessment; EPA = eicosapentaenoic acid; GLA = γ-linolenic acid; DHA = docosahexaenoic acid. Data are presented as medians (interquartile range, 25th to 75th percentiles), and *P *values were calculated using the Wilcoxon rank-sum test. ^b^Distributions considered non-normal on the basis of the Shapiro-Wilk test.

The incidence of diarrhea and vomiting was also evaluated. A total of two patients in the study group (3.8%) and three patients in the control group (5.7%) experienced vomiting. In addition, four patients in the study group (7.5%) and seven patients in the control group (13.2%) had uncontrolled diarrhea. The differences between the two groups were not considered statistically significant in terms of either vomiting (*P *= 0.74) or diarrhea (*P *= 0.43). During this study, no serious adverse events were recorded.

### Primary outcome: development of severe sepsis and septic shock

**Figure 2:**
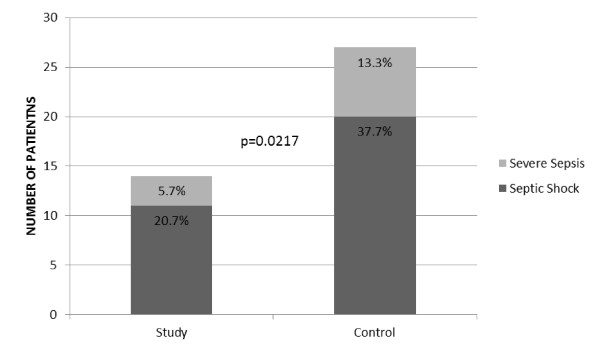
**Graph showing incidence of severe sepsis and septic shock**.

The population nourished with EPA/GLA developed less severe sepsis and septic shock than the patients nourished with the control diet. The ITT analysis of the primary outcome including all 115 patients demonstrated that more patients in the control group than in the study group evolved to severe sepsis and/or septic shock (29 patients (50%) in the control group versus 15 patients (26.3%) in the study group; *P *= 0.0259), with 3 patients (5.3%) developing severe sepsis and 12 patients (21.0%) developing septic shock in the study group versus 8 patients (13.8%) developing severe sepsis and 21 patients (36.2%) evolving to septic shock in the control group. Considering only the evaluable patients (*n *= 106), a total of 26.4% of the patients in the study group evolved to more severe forms of sepsis as compared to 51% in the control group (*P *= 0.0217) (see Figure 2). In the study group, in 5.7% of the patients, their conditions evolved to severe sepsis, and in 20.7% the patients' condition evolved to septic shock. In the control group, 13.3% of the population developed severe sepsis and 37.7% developed septic shock.

**Figure 3:**
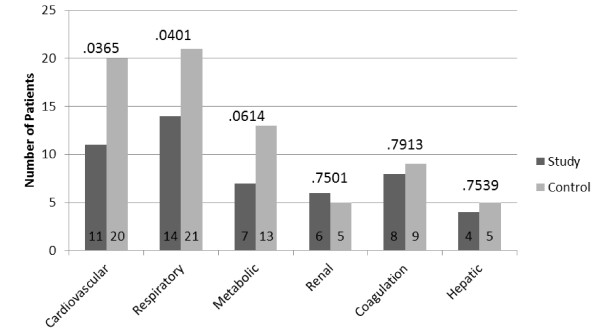
**Graph showing the development of individual system failures**.

With regard to the development of individual dysfunctions, it is notable that the observed difference is due basically to more development of cardiovascular and respiratory failure in the control group. The ITT analysis demonstrated that more patients in the control group than in the study group developed respiratory failure (23 patients (39.6%) versus 14 patients (24.6%), respectively; *P *= 0.0362) and cardiovascular failure (21 patients (36.2%) versus 12 patients (21.0%), respectively; *P *= 0.0381), with no significant differences observed in terms of coagulation failure (eight patients in the study group versus nine in the control group, respectively; *P *= 0.81), renal failure (six patients in the study group versus six in the control group, respectively; *P *= 0.63), metabolic failure (7 patients in the study group versus 13 in the control group, respectively; *P *= 0.07) or hepatic failure (four patients in the study group versus five in the control group, respectively; *P *= 0.74). Considering only the evaluable patients (*n *= 106), 11 patients in the study group developed cardiovascular failure as compared to 20 patients in the control group (*P *= 0.03). Additionally, 14 patients in the study group developed respiratory failure compared to 21 patients in the control group (*P *= 0.04). No significant differences were found in terms of metabolic, renal, hepatic or coagulation failure (Figure 3).

### Secondary outcomes

With regard to the results for all included patients, three patients required noninvasive mechanical ventilation at baseline, including one in the study group and two in the control group (see Table 3), although they did not fulfill the criteria for respiratory failure (all included had PaO_2_/FiO_2 _ratios > 300). The ITT analysis demonstrated that invasive mechanical ventilation was required by more patients in the control group than in the study group (20 patients (34.5%) versus 10 patients (17.5%), respectively; *P *= 0.295) and for a median of 7 days (interquartile range (IQR) 4 to 12) days in the study group and 15 days (IQR 8 to 21) in the control group (*P *= 0.003). Patients in the control group had fewer ICU-free days than the study group (means ± SD: 14.7 ± 5.1 days versus 21.1 ± 4.7 days, respectively; *P *< 0.001) as well as fewer hospital-free days than the study group (means ± SD: 10.3 ± 8.6 days versus 19.5 ± 7.8 days; *P *< 0.001). The analysis of the 106 evaluable patients showed that more patients in the control group than in the study group required invasive mechanical ventilation (18 patients in the control group versus 10 patients in the study group; *P *= 0.0394), but no differences were found in terms of noninvasive mechanical ventilation (five patients in the study group versus six patients in the control group; *P *> 0.05). The patients nourished with EPA/GLA required mechanical ventilation for a shorter time than the control patients (median 7 days (IQR 4 to 7) in the study group versus 15 days (IQR 9 to 21) in the control population; *P *= 0.003). In addition, patients who were fed the study diet remained in the ICU for fewer days (median 7 (IQR 4 to 12) days in the study group versus 13 days (IQR 9 to 18) in the control population; *P *< 0.0001), as well as fewer days at the hospital (9 days (IQR 6 to 14) in the study group versus 19 days (IQR 13 to 24) in the control group; *P *< 0.0001) (see Table 5). Differences were also significant when expressed in terms of ICU-free and hospital-free days. The mean total ICU-free days were 20.8 ± 3.9 in the population nourished with EPA/GLA versus 14.3 ± 5.1 in the control population (*P *< 0.001). The mean total hospital-free days were 19.1 ± 6.3 in the study group and 10.2 ± 8.2 in the control group (*P *< 0.001).

**Table 5:** Use of hospital resources^a^

Variable	Study (*n *= 53)	Control (*n *= 53)	*P *value
Mechanical ventilation support, *n *(%)			
Invasive	10 (18.9%)	18 (34%)	0.03984
Noninvasive	5 (9.4%)	6 (11.3%)	0.7501
Duration, days			
Mechanical ventilation support^b^	7 (4 to 12)	15 (9 to 21)	0.0033
ICU stay^b^	7 (4 to 12)	13 (9 to 18)	< 0.0001
Hospital stay^b^	9 (6 to 14)	19 (13 to 24)	< 0.0001

^a^Data are presented as medians (interquartile range, 25th to 75th percentiles), and *P *values were calculated using the Wilcoxon rank-sum test. ^b^Distributions considered non-normal on the basis of the Shapiro-Wilk test.

No patients requiring vasopressors or inotropic drugs were included at baseline. During the 28-day follow-up period, dobutamine was required for 9 patients (17%) in the study group and 13 patients (24.5%) in the control group (*P *= 0.34), with similar daily doses used for both groups (9.96 μg/kg/minute for the study group and 9.57 μg/kg/minute for the control group; *P *= 0.77). Dopamine was used to treat 12 patients (22.6%) in the study group and 17 patients (32.1%) in the control group (*P *= 0.28), with mean daily doses of 23.4 μg/kg/minute in the study group and 21.5 μg/kg/minute in the control group (*P *= 0.87). Norepinephrine was used to treat 9 patients (17%) in the study group and 11 patients (20.7%) in the control group (*P *= 0.61), with mean daily doses of 2.2 μg/kg/minute in the study group and 1.8 μg/kg/minute in the control group (*P *= 0.81).

**Figure 4:**
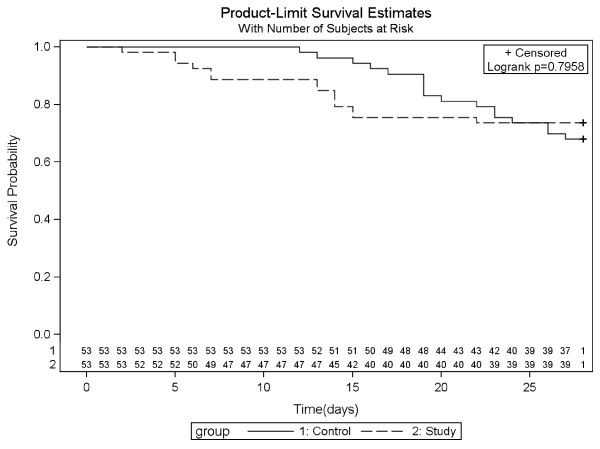
**Kaplan-Meier survival curves graphed for 28-day all-cause mortality**.

No differences were found in the ITT analysis of 28-day all-cause mortality (*n *= 115). By day 28, 15 of 57 patients in the study group had died and 16 of 58 patients in the control group had died (*P *= 0.72). Similarly, no difference was found in the 28-day all-cause mortality of the evaluable patients (*n *= 106) (Figure 4), among whom 39 patients (73.5%) in the study group and 37 patients (69.8%) in the control group were alive after 28 days. The log-rank test analysis indicated that the difference was not significant (*P *= 0.79).

## Discussion

Currently available strategies for the treatment of patients with sepsis focus on the management of the more severe forms of the disease (severe sepsis and/or septic shock), when the patients already have multiple-system organ dysfunctions, which is always associated with elevated death risk [30,31]. The contradiction is that there is also a consensus that, when treating patients with sepsis, most, if not all, available strategies are time-dependent [32].

Unfortunately, information about possible strategies to be applied in the early stages of sepsis is extremely limited, as is the number of trials evaluating possible therapies this early in the disease time line. The main purpose, and probably the most interesting characteristic of the present trial, was to apply a nutritional strategy that has proven to be of great value in the management of patients with late sepsis-associated respiratory failure [1], and is associated with an important reduction in the development of new organ failures [20], early in the evolutionary stage of sepsis when no organ dysfunction has been identified and to test whether this nutritional strategy can be used to help slow the progression of the disease.

In this study, we found an association of the use of an EPA/GLA diet with reduced incidence of severe sepsis and/or septic shock. Although the mechanisms underlying the results of the present trial are unclear, there are several possible roles associated with EPA, GLA and antioxidants which, acting alone or together, may help us to understand how this diet can lead to the observed effects, among them being the regulation of AA levels in the inflammatory cell membranes, downregulation of NF-κB, lower production of inflammatory eicosanoids, antioxidant-mediated reduction in overall reactive oxygen species levels and a quicker recovery from inflammation due to production of resolvins and protectins, among several others.

In this trial, we used a control diet different from those used in previously published works [16-18]. Our control diet was not enhanced with lipids but was higher in carbohydrates and was considered a standard ICU diet. The reason for our use of that control diet was that one major focus of criticism of previous work is precisely the control diet that was used (Pulmocare; Abbott Nutrition). Maybe the main reason for such criticism is that a high-lipid enteral diet is not considered by many to be a standard diet for critically ill patients. It is a common misunderstanding that enteral formulas enriched with Ω-6 lipids such as linoleic acid (LA, or 18:2n6; for example, from corn oil) can produce upregulation of the inflammatory response and, for that reason, such formulas may not be used to feed patients with hyperinflammatory diseases. Production of AA from LA involves three key enzymatic steps: Δ-6-desaturation to form 18:3n6 (GLA), followed by elongation to 20:3n6 (dihomo-GLA (DGLA)) and a Δ-5-desaturation step to produce 20:4n6. Thus, LA-enriched formulas cannot worsen inflammation simply because the activity of the enzymes Δ-5-desaturase and Δ-6-desaturase are severely compromised during critical illness by the release of stress and catabolic hormones (for example, glucocorticoids and catecholamines) [33,34] limiting the ability to form AA despite the provision of LA [35]. GLA is a metabolite of LA that can bypass the decreased expression of Δ-6-desaturase. Its elongation product, DGLA, is incorporated into the inflammatory cell membranes, and the formation of DGLA suppresses leukotriene biosynthesis and can be metabolized to form prostaglandin E_1_, a potent pulmonary vasodilator [36,37]. DGLA is also metabolized by 5-lipoxygenase to form 15-hydroxyeicosatrienoic acid, which inhibits the formation of leukotriene B_4_. In fact, an enteral diet enriched with GLA cannot increase AA levels in immune cell membranes [38] and, on the other hand, can increase anti-inflammatory activity by incorporating DGLA into the immune cell membranes. Despite all of the above-mentioned evidence, the previous criticism of other studies highlights the importance of this trial as the first one to demonstrate a positive result in terms of primary and secondary outcomes comparing the EPA/GLA diet with a standard ICU formula.

In this study, we report a reduction in both ICU and hospital LOS for patients fed a diet with EPA/GLA compared to the control population. The differences represent a mean of 6.5 additional ICU-free days and 8.9 additional hospital-free days associated with the use of the study diet. These results are in accord with previously published studies [16-18] in which EPA/GLA diets were used to treat critically ill patients requiring mechanical ventilation, all of which reported reductions in ICU and hospital LOS. In fact, a recent meta-analysis [19] associated the use of EPA/GLA with a mean of 4.3 more ICU-free days compared to patients fed a control diet. Sepsis represents an important financial burden on the healthcare system [39,40], and any reduction in terms of LOS must be considered to have a potential economic impact regarding reductions in the overall cost of care.

It is important to note that the reported number of patients requiring mechanical ventilation (invasive or noninvasive) was slightly different in the study groups with regard to the number of patients considered to have developed respiratory failures since the end of the study. The reason for that finding was that, during the 28-day follow-up period, one patient in the study group and three patients in the control group required noninvasive mechanical ventilation but did not fulfill the PaO_2_/FiO_2 _ratio criterion for respiratory failure.

Previously published works [17,18] and the present study have many relevant characteristics in common. They all used a study diet containing carbohydrates and proteins together with high levels of EPA/GLA. They all started enteral nutrition as early as possible and delivered the diet continuously using an enteral feeding pump. In a recent study (the EDEN-Omega Study) conducted by the EDEN-Omega NHLBI ARDS Network, investigators reported different findings when treating critically ill patients [41]. They found no benefit in using a module containing EPA/GLA to treat patients requiring mechanical ventilation, and the study was stopped because of futility. Comparison of the EDEN-Omega Study with the previously published works can lead to dangerous misinterpretation of the current evidence. First and foremost, the patients enrolled in the EDEN-Omega study received a bolus of EPA/GLA twice daily, not an enteral formula containing EPA/GLA as part of it. It is uncertain whether these pharmaconutrients act differently if not provided using continuous feeding and as part of an enteral nutrition formula. Absorption of individual macronutrients (such as lipids) at the intestinal level can be drastically affected by the presence or absence of other nutrients. Additionally, it has been demonstrated [42] that the delivery of an EPA/GLA diet by continuous feeding can produce important changes in the production of inflammatory mediators associated with modulation of plasma phospholipid levels. It is not clear whether a bolus of EPA/GLA given twice daily can provide similar changes in plasma phospholipids. Unfortunately, to date, the EDEN-Omega NHLBI ARDS Network investigators have not made publicly available the plasma phospholipid measurements of the patients included in the EDEN-Omega study, data that are of pivotal importance to allow a fair trial comparison between their trial and those previously published. Moreover, the EDEN-Omega study did not control for several other variables, including the different levels of other macronutrients (such as proteins) in the underlying diets fed to their patients, producing a huge source of bias in their data. Finally, the EDEN-Omega study was not designed as an early intervention trial, but was in fact an early versus late intervention study, whereas early enteral feeding was described as a key factor in all previous trials. The currently available nutrition guidelines continue to unanimously recommend the use of an EPA/GLA diet in critically ill and mechanically ventilated patients with ALI/ARDS [1,20]; therefore, not to use this strategy in the indicated population of patients merely on the basis of the results of an unpublished single trial in which so many important questions remain to be answered does not appear to be justifiable.

Interaction between fish oil and sepsis is very complex and variable, depending on the dose administered and probably the route of administration as well. Most of the available evidence associated with the benefits of fish oil was produced when this active pharmaconutrient was used as part of enteral nutrition formulations. However, the number of trials testing parenterally administered fish oil in a variety of clinical situations, including sepsis, is growing [43,44]. For instance, in a recently published study using a fish oil-based lipid emulsion in patients with sepsis, Barbosa and co-workers [45] demonstrated significant improvements in the PaO_2_/FiO_2 _ratio that were similar to the effects reported with the use of enteral nutrition.

Although in the present study we have demonstrated a reduction in the development of cardiovascular and respiratory failure in the patient population nourished with EPA/GLA as compared to the control population, this was not associated with a significant reduction in mortality. The probable reason for this finding is that this study included patients with early sepsis and no organ dysfunction, a clinical situation usually associated with lower mortality rates than are found in patients with severe sepsis and/or septic shock [46]. The sample size of this trial was not calculated to demonstrate mortality differences, and this particular variable should be evaluated in future studies.

Some important limitations need to be considered when evaluating the results of the present study. One of the most important is that this study included only those patients in need of enteral nutrition. During the enrollment of the patients, the investigators considered this to be very relevant and to represent a limitation in terms of the number of potential patients who could be included in this trial, since most patients whom we found to be in the early stages of sepsis did not require enteral nutrition, being able to receive oral feeding. In addition, the study included only patients at the ICU, which reduced even more the number of potential patients to be included, since the majority of the patients with early sepsis are usually not at the ICU but in the general wards. As a result of these important limitations, we believe that the population of patients included in this study was somehow selected, being constituted by elderly patients, most of whom were already receiving treatment and enteral feeding at their homes because of previous limitations such as stroke and eventually developing diseases such as community-acquired pneumonia, therefore requiring hospital treatment. In addition, this study excluded patients with a BMI ≥ 29, and for this reason generalization of the results to the obese population of patients is not possible. Finally, it was not possible to report changes in the Δ or component SOFA scores, since this trial was not designed to collect daily SOFA scores. Therefore, it is uncertain whether the observed reduction in the development of severe sepsis and/or septic shock is associated with reductions in the SOFA score over time. This association represents an important variable to be evaluated in future trials in which an EPA/GLA diet is fed to septic patients.

It will be important to produce further evidence and reproduce the present work in a more broad population of patients in the early stages of sepsis using an EPA/GLA diet, maybe as an oral supplement, and not delivering these pharmaconutrients only when an enteral feeding tube is in place.

## Conclusions

The present study presents evidence that enteral nutrition with EPA/GLA and elevated antioxidants, when used in the early stages of sepsis in patients without any organ failure and in need of enteral nutrition, can play a beneficial role by slowing the progression of the disease to severe sepsis and septic shock. The possible benefit of EPA/GLA supplementation in patients receiving oral feeding needs further evaluation.

## Key messages

• Although EPA/GLA is currently recommended for critically ill, mechanically ventilated patients with ALI/ARDS in accordance with international guidelines, there is no evidence that this diet has any benefit when used in the early stages of sepsis.

• This report describes a prospective, multicenter, double-blinded, controlled study designed to evaluate the effects of enteral nutrition with EPA/GLA in critically ill patients with early sepsis without organ failure.

• When used in the above-mentioned patient population, enteral nutrition enriched with EPA/GLA was associated with less development of severe sepsis and/or septic shock, primarily because of the reduced number of cardiovascular and respiratory failures in the study population.

• An additional benefit was found in terms of reduced hospital and ICU LOS, which represents an important reduction in the overall cost of care for the treatment of this patient population.

• No effect was observed with regard to 28-day all-cause mortality.

## Abbreviations

ALI: acute lung injury; APACHE II: Acute Physiology and Chronic Health Evaluation II; ARDS: acute respiratory distress syndrome; BEE: basal energy expenditure; BMI: body mass index; CI: confidence interval; DHA: docosahexaenoic acid; EPA: eicosapentaenoic acid; GLA: γ-linolenic acid; IQR: interquartile range, 25th to 75th percentiles; ITT: intention to treat; LOS: length of stay; MODS: Multiple Organ Dysfunction Score; NF-κB: nuclear factor κB; OR: odds ratio; SGA: Subjective Global Assessment; SOFA: Sequential Organ Failure Assessment.

## Competing interests

APA received speaker honoraria and a research grant from Abbott Nutrition International. MM received honoraria and participated in events sponsored by Pfizer and Novartis. LFM, SML, AMI, DT, ER and EBM have no conflicts of interest associated with the present work.

## Authors' contributions

APA was responsible for the study design and trial registration, participated in the coordination of the study and wrote the manuscript. LFM, SML, AMI, DT, ER, MM and EBM collaborated in the collection of data for this study and in the revision of the manuscript. All authors read and approved the final manuscript.
